# Calendar

**DOI:** 10.1155/S1463924686000305

**Published:** 1986


Journal of Automatic Chemistry/*Journal of Clinical Laboratory Automation*, Vol. 8, No. 3 (July–September 1986), p. 155

### Calendar

### SEPTEMBER

23 to 25 September *Analyticon 86: Commonwealth Institute, London.* Contact Scientific Symposia Ltd, 33–35 Bowling Green Lane, London EC1R 0DA.

### OCTOBER

23 to 25 October *Annual Joint Meeting of the German, Austrian and Swiss Societies of Clinical Chemistry with the Société Française de Biol. Clinique: Basel, Switzerland.* Contact Dr Peter R. Huber, Laboratorien FK, Kantonsspital Basel, CH 4031 Basel, Switzerland.

### NOVEMBER

11 to 12 November *Symposium on Control and Prevention of Runaway Chemical Reaction Hazards: Hotel Okura, Amsterdam, The Netherlands.* Contact Fiona Spindlove, IBC Technical Services Ltd, Bath House, 56 Holborn Viaduct, London EC1A 2EX.

### 1987

### MARCH

9 to 13 March *38th Pittsburgh Conference and Exposition on Analytical Chemistry and Applied Spectroscopy: Convention Center, Atlantic City, New Jersey, USA.* Contact Mrs Alma Johnson, Program Secretary, 12 Federal Drive, Suite 322, Pittsburgh, Pennsylvania 15235, USA.

### 1988

### APRIL

18 to 21 April *Flow Analysis IV: Las Vegas, Nevada, USA.* Contact Professor Gilbert Pacey, Department of Chemistry, Miami University, Oxford, Ohio 45056, USA.

*Conference organizers should send full details of their meeting for inclusion in 'Journal of Automatic Chemistry's' calendar at least three months before the event. Information to the Editor, Journal of Automatic Chemistry, Taylor & Francis Ltd, 4 John Street, London WC1N 2ET.*

### SCIENTIFIC COMPUTING AND AUTOMATION (EUROPE)

*Amsterdam: 13 to 15 May 1987*

This will be Europe's first conference and exhibition specifically for computing and automation in the sciences. Scientific Computing and Automation (Europe) will be the European counterpart to its successful sister meeting in the USA carrying the same name.

SCA Europe will be supported by a number of European societies, among them the Royal Society of Chemistry, and will be held at the RAI Congress Centre in Amsterdam, The Netherlands, between 13 and 15 May 1987. A full scientific programme will form the backbone of the meeting; the programme will be set up by a distinguished scientific board under the chairmanship of Professor D. L. Massart from the Vrije Universiteit of Brussels, Belgium.

A number of workshops and short courses will also be run in conjunction with the conference.

SCA Europe will devote both its interdisciplinary and scientific parallel sessions to the latest advances in computing and automation in the scientific environment.

These include chemistry (pharmaceutical, analytical, clinical, synthesis), the life sciences (clinical microbiological), engineering and technology (biomaterials, chemical) and physics (mainly applied geophysics).

Within the framework of these three specialist fields, the international scientific community will be able to share their experiences in:

Computing (for example artificial intelligence, expert systems, image analysis, Computer Aided Learning (CAL), pattern recognition, molecular graphics, databases, simulation and off-the-shelf software).
Automation (for example LIMS, networking, data acquisition, sensors, process control).
Robotics (laboratory products).

All the plenary and disciplinary sessions will be chaired by specialists in their own field who will also sit on the scientific program commitee and thus ensure the high quality of submitted papers. Papers should be sent to: Professor D. L. Massart, Vrije Universiteit of Brussels, Laarbeeklaan 103, B-1090 Brussels, Belgium.

*Exhibitors and registrants should contact K. Foley, Scientific Computing and Automation, PO Box 330, 1000 AH Amsterdam, The Netherlands. Tel.: 020 5862 828.*

